# Autophagy in alzheimer disease pathogenesis and its therapeutic values

**DOI:** 10.1080/27694127.2025.2471677

**Published:** 2025-05-08

**Authors:** Gabrielle Angst, Nuo Jia, Luis E. Tron Esqueda, Yanbo Fan, Qian Cai, Chenran Wang

**Affiliations:** a Department of Cancer Biology, University of Cincinnati College of Medicine, Cincinnati, OH, USA; b Department of Cell Biology and Neuroscience, Division of Life Science, School of Arts and Sciences, Rutgers, The State University of New Jersey, Piscataway, NJ, USA; c Department of Pharmacology and System Physiology, University of Cincinnati College of Medicine, Cincinnati, OH, USA; d Department of Internal Medicine, Division of Cardiovascular Health and Disease, University of Cincinnati College of Medicine, Cincinnati, OH, USA

**Keywords:** Alzheimer disease, amyloid-β, autophagy, blood-brain-barrier, glia, neurofilament tangles, neuroinflammation, neuron

## Abstract

Alzheimer disease (AD) is the most common form of dementia with hallmarks of β-amyloid deposits, neurofilament tangles, synaptic loss and neuronal death in the patient’s brain. AD is a heavy burden in an ageing society as there are no effective therapies in treating the causes or slowing down its progression. Autophagy is a conserved process through formation of double membrane structure, namely autophagosome which is delivered to lysosome to digest cellular disposals. Autophagy maintains homoeostasis in the brain and is generally considered to protect brain functions against ageing. The first evidence of autophagy involvement in AD is that there is decreased expression of autophagy essential genes in post-mortem AD brains. Autophagy is also believed to be protective in neurodegeneration. However, the molecular and cellular mechanisms for dysfunction of autophagy in AD are not fully understood. Recent studies of autophagy regulation in AD cover the findings not only in neurons, but also from fast growing evidence for their importance in glia and brain vascular system. Thus, this review composes pertinent information regarding the involvement of autophagy in neurons, glias (including microglia, astrocyte, and oligodendrocyte), and brain vascular cells in AD, and their unique cellular mechanisms of this connection in AD pathology. We will provide effectual insights both in investigating autophagy in AD pathological mechanisms and in establishing a strategic approach for developing autophagy-based AD therapies.

## Introduction

Alzheimer disease (AD) is a neurodegenerative disease and the most common form of dementia, estimated to be 60-70% of the cases [1]. This disease affects 6.7 million American people and more than 55 million people worldwide in 2023 [2]. AD is clinically characterised by progressive cognitive decline, memory loss, and loss of motor skills. The pathology of AD is a complex muti-factorial one which includes neuropil threads, neuronal loss, and synapse loss as well as misfolding of the amyloid beta (Aβ) protein and the Tau protein, as well as the sustained neuroinflammation [3,4]. In most cases of AD, pathological symptoms will be present for years before any clinical symptoms can be observed. AD occurs in two forms, early onset Alzheimer’s disease, also known as familial AD (fAD), and late onset Alzheimer’s disease (LOAD), with LOAD being the much more common variation for more than 90% of the patients. Between the two subtypes, fAD will usually progress more aggressively. In fAD, the mutations of genes for Aβ plaques generation are believed to be the main factor contributing to its pathogenesis. Aβ plaques are principally composed of misfolded APP (β-amyloid precursor protein). The APP is a single-pass transmembrane protein highly expressed in the brain and metabolised by β-secretase (BACE1) and γ-secretase. Mutations in Presenilins (presenilin1, PS1 and presenilin2, PS2), the catalytic components of γ-secretases, are responsible for approximately 40% of fAD monogenic cases. The physiological function of BACE1 and γ-secretase can be linked to synaptic scaling [5] and synaptic vesicle release [6]. However, despite those functions for APP procession, the full spectrum of functions that APP plays in the physiological conditions remains to be fully explored. Mutations of APP gene cluster around the γ-secretase cleavage site and cause a change in amino acids adjacent to the BACE1 cleavage site. These mutations are responsible for an increase in the production of the less soluble and more toxic Aβ42 [7]. In contrast to fAD, the cause for LOAD is still largely unknown. Several genetic risk factors have been identified through genome-wide association study, including mutations in Apolipoprotein E (*Apoe*) and Triggering receptor expressed on myeloid cells 2 (*Trem2*), which highly express in astrocyte and microglia. Currently, the exact mechanisms that underlie LOAD are not fully understood and there are no known effective treatments or ways to prevent its onset.

Autophagy is a highly conserved catabolic process within eukaryotic cells, involving the breakdown of organelles, proteins, macromolecules, and intracellular pathogens to maintain cellular homoeostasis [8,9]. Autophagy continuously occurs at low levels under normal circumstances in the brain and its activity will be upregulated under stress conditions including environmental, metabolic, toxic, and injury circumstances [9]. The materials degraded from this process are then recycled to be used for building materials or energy resources. Having dysfunctional autophagy has been linked to diseases such as cancer, infection, metabolic diseases, and neurodegenerative disorders [10-12]. There exist three main types of autophagy, namely macroautophagy, microautophagy, and chaperone-mediated autophagy (CMA), with each ultimately delivering cargo to lysosomes for degradation. Macroautophagy involves the sequestration of cytoplasmic cargo into double membraned vesicles called autophagosomes [13,14]. These autophagosomes then deliver the content to lysosomes for degradation by acidic hydrolases. CMA is a selective form of autophagy where chaperone protein Hsc70 recognises specific cargo and then transports it to proteins along the lysosome’s surface where the channels move the cargo inside lysosome for degradation [15,16]. What distinguishes microautophagy is that it involves the direct uptake of material into lysosomes without an intermediate [17,18]. Of the three types of autophagy, microautophagy is the least understood while macroautophagy is the most studied. In the following discussion, we will focus on macroautophagy (herein as autophagy) for its mechanisms and roles in AD.

**Figure 1.**
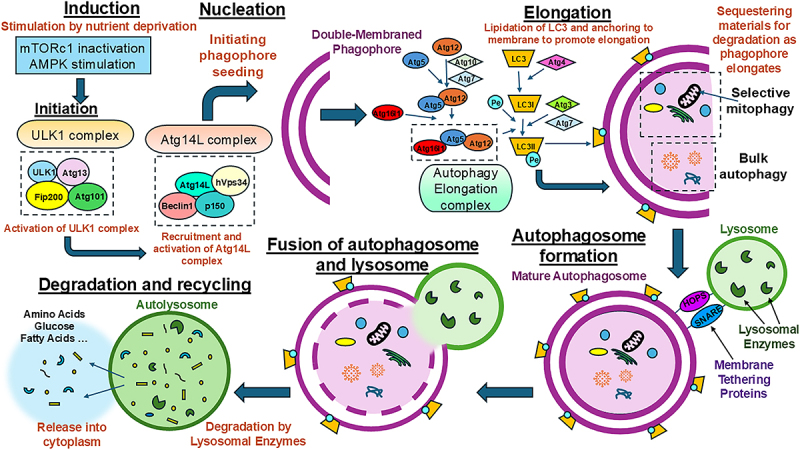
Autophagy machinery in mammalian cells. Autophagy can be induced by mTORC1 inactivation via nutrient deprivation or through stimulation of AMPK after energy stress. The induction step is mediated by the activation of the ULK1 complex, which is composed of ULK, ATG13, RB1CC1, and ATG101. The ULK1 complex recruits the assembly of the ATG14L complex, made up of BECLIN 1, ATG14L, P150, and hVPS34. The ATG14L complex then takes part in nucleation, which is the assembly of molecules, mainly autophagy related proteins (ATG), to the phagophore assembly site. This allows for the initial seeding of the phagophore that is responsible for sequestering material for degradation. The autophagy elongation complex is composed of ATG5, ATG12, and ATG16L1 and this complex helps in the lipidation of LC3. Lapidated LC3-II will then bind to the growing phagophore membrane and this association aids in completing autophagosome formation. Once complete, the autophagosome will fuse with a lysosome with the help of SNARE and the homotypic fusion and protein sorting (HOPS) tethering complex. Once fused, an autolysosome is created where the materials will be degraded by lysosomal enzymes. After degradation, the final materials are released as fatty acids, amino acids, and glucose to be reused by the cell.

Autophagy is a multi-step process consisting of autophagosome forming steps of induction, initiation, elongation, closure and maturation, as well as autophagosome fusion with lysosomes and ultimately the degradation of autophagosome (Figure 1). The classic induction of autophagy can be stimulated through nutrient deprivation. Mechanistic target of rapamycin complex 1 (mTORC1) is a master regulator for cell metabolism and it senses the changes of nutrient levels in the environment [19,20]. mTORC1 inactivation by nutrient deprivation stimulates the autophagy induction complex of ULK1 (Unc-51-like kinase 1 complex), RB1CC1 (RB1 inducible coiled-coil 1), ATG13 (autophagy related gene 13) and ATG101 to form a double-membraned phagophore [21,22]. The phagophore then expands and elongates under the activation of Beclin1/Becn1complex, corralling the cytoplasm and organelles meant for subsequent degradation. This step uses WD repeat domain, phosphoinositide interacting 2 (WIPI2)-mediated recruitment of the ATG12-ATG5-ATG16L1 complex, which enhances the cytosolic form of MAP1LC3-I (LC3-I) conjugated to phospholipid phosphatidylethanolamine (PE) to form LC3-PE conjugate (LC3-II). This lipidation will anchor LC3-II to the autophagosome so that it can function in the elongation and closure of mature autophagosome [20]. Once a mature autophagosome is formed, it will fuse with the lysosome to become an autolysosome. For this process, the ATG proteins are replaced by soluble NSF attachment protein receptors (SNAREs) and homotypic fusion and protein sorting complex. The cargo in autolysosome is degraded by lysosomal enzymes and then released as materials such amino acids, lipids, and glucose [23]. The process of autophagy can be divided into two broad categories, non-selective one and selective one. Non-selective autophagy involves the transport and degradation of cargo indiscriminately while selective autophagy is used for specific degradation of certain materials such as pathogens (xenophagy), mitochondria (mitophagy), misfolded or aggregated proteins (aggrephagy), and so on [24].

With the progress of AD research, people now understand that the pathology of this disease is not only involved with abnormal protein aggregates and neuronal death, but it also has significant impact on the environment involved with glias and the brain endo-vascular systems. Autophagy level is diminished in AD brain and abolishing autophagy has significant impact on the acceleration of AD progression. In this review, we combine our current understanding of autophagy in major brain cell types, including neurons, glia, and endothelial cells to summarise and discuss the mechanism and impact of autophagy in AD and its potential application as a target to relieve AD symptoms or slow down its progression.

## Autophagy of neuron in AD

### Autophagy and its physiological roles in neurons

Neurons are highly polarised cells and are composed of a complex dendritic arbour and a long axon that emerges from the soma and can extend more than a metre in the human body. Given the post-mitotic nature of neurons, sophisticated mechanisms that allow not only the efficient delivery and constant supply of newly synthesised proteins and organelles, but also timely and spatial disposal of damaged proteins and dysfunctional organelles, are of great importance for neuronal function and survival [25-27]. As an evolutionarily conserved pathway that functions as a key mechanism for maintaining protein and organelle physical and functional integrity, autophagy has been shown to play an essential role in neuronal homoeostasis to prevent neurodegeneration. About two decades ago, an initial study of neuronal autophagy reported that loss of autophagy function in neurons is fatal within the first postnatal months of rodents [28]. Follow-up studies further revealed marked neuronal death in the cerebral cortex coupled with abnormal neuronal accumulation of polyubiquitinated proteins as inclusion bodies in mice with selective deletion of *atg7* or *atg5* in neurons (pan neural nestin-cre) [29,30]. In addition, several models targeting different neuronal types with autophagy deficiency have been developed, including targeted knockout of *atg5* or *atg7* in Purkinje neurons of the cerebellum [31,32], of *atg7* in agouti-related peptide neurons of the hypothalamus [33], and of *atg5* or *atg7* in rhodopsin neurons of the retina [34,35]. Besides cell-autonomous Purkinje neurodegeneration, *atg5* or *atg7*-deficient mice in Purkinje neurons exhibited axonal swellings, the earliest sign of homoeostatic disruption preceding progressive dystrophy and degeneration of axons [31,32]. Additional work in the cellular model systems demonstrated that the capacity of autophagic clearance varies in different neuronal types [36], which is in accord with the distinct sensitivity of individual neuronal types to aggregate-prone protein accumulation. Results from these studies collectively indicate that autophagy is necessary for neuronal maintenance, while its deficiency is detrimental to neurons.

Neuronal processes are decorated with many synapses in which neurotransmission comes along with high energy demands, fast protein and organelle turnover rates, and excessive membrane exchange and renewal [37-39]. Anatomically, synapses are located far away from the soma of neurons. Emerging evidence implicates autophagy as a key mechanism that has adapted to the microenvironment of synapses to sustain their function and homoeostasis. For example, neuronal autophagy regulates presynaptic transmission by controlling the endoplasmic reticulum (ER) in axons [40]. Defects in neuronal autophagy were shown to cause axonal ER accumulation and increased excitatory neurotransmission. Basal autophagy is also necessary for maintaining presynaptic structure and function in dopaminergic neurons [41]. Loss of autophagy core component Atg7 in these neurons led to abnormally enlarged presynaptic terminals, accompanied by enhanced neurotransmitter release and faster presynaptic recovery. Unexpectedly, studies using autaptic hippocampal cultures failed to demonstrate any effect of basal autophagy on neurotransmission [42]. While the significance of basal autophagy might vary at the terminals of different neuronal types, autophagy-mediated synaptic cargo clearance is most likely crucial under conditions when demands for protein/organelle turnover are robustly increased.

It is worth noting that many studies also provide striking evidence showing (neuro)protective effects upon upregulation of autophagy function in different model systems. Increasing basal autophagy activity in *Caenorhabditis elegans* (*C. elegans*) results in an extended lifespan, attributable to multiple distinct but overlapping mechanisms, including nutrient restriction, altered mitosis, and mitochondrial renewal [43-45]. In *Drosophila*, the pan-neuronal elevation of Atg8a was reported to lead to longevity [46]. In line with these observations in *C. elegans* and *Drosophila* models, lifespan extension was observed in mice with overexpression of Atg5, accompanied by anti-ageing phenotypes, such as leanness, increased insulin sensitivity, and improved motor function [47]. Taken together, these findings demonstrate the protective effect of autophagy potentiation against ageing and suggest that autophagy may operate as a critical checkpoint for controlling protein and organelle quality/function, thus constituting a pivotal homoeostatic mechanism in neurons.

### Autophagy mechanism in neurons

#### Autophagosome biogenesis

**Figure 2.**
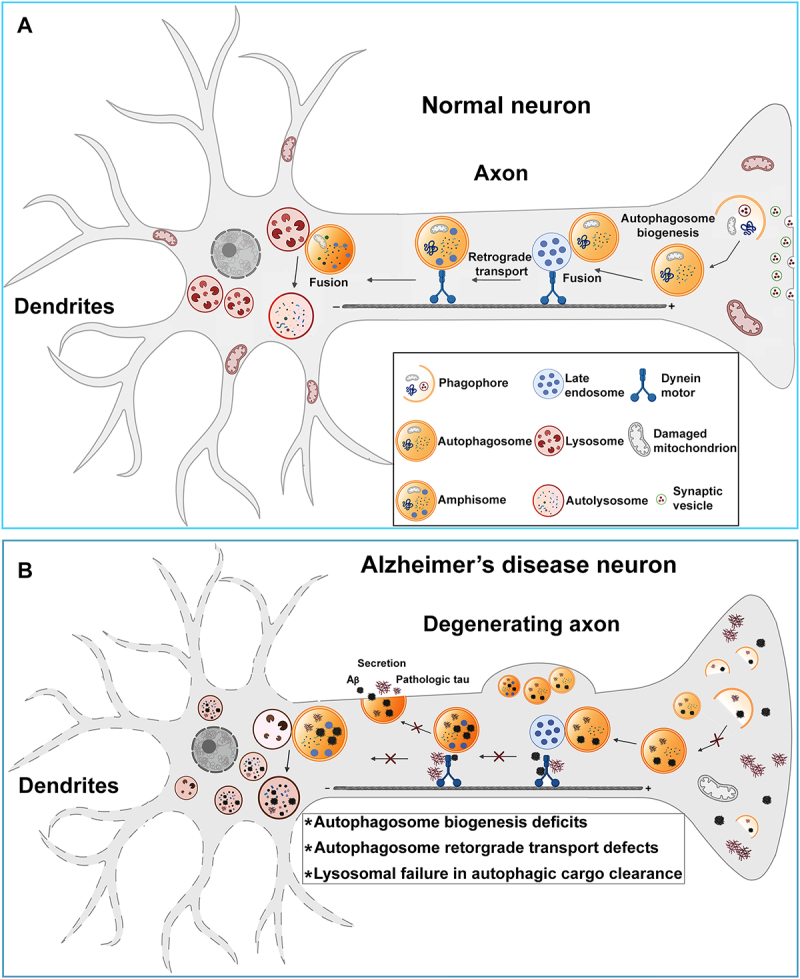
Autophagy in normal and AD neurons. A. In neurons, (macro)autophagy mainly occurs in axons and synapses and is initiated by forming a double-membrane structure, phagophore. Damaged cytosolic components and dysfunctional organelles targeted for lysosomal degradation are sequestered within this structure during phagophore expansion and enclosure to form an autophagosome. Newly generated autophagosomes rapidly acquire retrograde motility by recruiting dynein motors through fusion with late endosomes as amphisomes. Thus, cargo-loaded autophagosomes travel from the distal axon to the soma, where autophagic cargos are eliminated within autolysosomes through the activity of lysosomal hydrolases. B. In AD neurons, while autophagosomes are an essential site for APP amyloidogenic processing and Aβ generation, autophagy dysfunction is also indicated, attributable to deregulations in the multiple steps of this pathway, including autophagosome formation deficiency, autophagosome retrograde transport impairment, and autophagic cargo clearance deficits owing to lysosomal failure, augmenting the build-up of Aβ and tau. Additionally, the autophagy-based extracellular secretion of Aβ and tau may be disrupted, thereby aggravating intraneuronal Aβ and tau accumulation/aggregation in AD neurons.

While autophagy has been extensively studied in many non-neural cell types, how this pathway functions in neurons remains far from clear. Most studies to date investigating neuronal autophagy have been focused on the mechanisms of compartmentalised autophagosome formation and maturation, the molecular machinery involved, and the processes of autophagic cargo selection and elimination within lysosomes, establishing that neuronal autophagy is a process with pronounced temporal and spatial specificity (Figure 2) [48-55]. Autophagy is known to be initiated with the formation of autophagosomes involving the sequential recruitment of multi-subunit complexes that accommodate the continuous growth of the phagophore membrane and LC3 lipidation (Figure 1) [10,56]. In neurons, autophagosome biogenesis is characterised by high temporal specificity, as individual autophagosome formation events appear to occur within minutes [49,50,57,58]. It is also important to note that autophagosomes are predominantly generated in the distal regions of neurons, including axons and synapses where autophagic cargos/substrates, such as dysfunctional cellular components and defective organelles, are sequestered within autophagosomes [49,50,57,58]. As a result, neurons constantly tackle unique spatial challenges given that the cargoes loaded in autophagosomes must be returned from distal axons through a long-distance retrograde transport process to the soma, where autophagic cargos can be eliminated within lysosomes. Moreover, unlike other cell types, autophagosome biogenesis is constitutively active in neurons. It remains steady under basal and growth-promoting conditions [48-50,57,58], supporting the view that autophagy acts as a baseline homoeostatic mechanism in neurons.

#### Autophagosome retrograde axonal transport

Once autophagosomes are produced in the axons and synapses of neurons, retrograde axonal transport is necessary to deliver these cargo-loaded autophagosomes to the soma for lysosomal clearance. Earlier work in the axons of cultured sympathetic neurons using light microscopy combined with correlative electron microscopy revealed retrograde movement of membranous organelles that appeared as multilamellar structures, implying that these organelles could be autophagic vacuoles [59]. More direct evidence depicting dynamic retrograde transport of autophagosomes in axons has been available lately from many studies using primary mouse embryonic cortical neurons [48] or dorsal root ganglion neurons from rats [50] or transgenic mice expressing autophagosomal marker LC3 fused to GFP [49]. In accord with the findings from these studies, autophagosome retrograde transport was proposed to be mediated by cytoplasmic dynein motor, a major microtubule-dependent and minus-end-directed motor [60,61]. As expected, mutation or inhibition of dynein motors leads to disruption of autophagic cargo clearance [61,62]. So far, multiple adaptors or effector proteins of dynein motors have been identified for their potential role in attaching dynein motors to autophagosomes, thus enabling autophagosome retrograde movement [50,63-68]. It is worth noting that two studies have provided initial compelling evidence demonstrating that newly formed autophagosomes acquire endosomal markers to form amphisomes, thereby allowing autophagosomes to gain their retrograde motility [48,50]. Our studies have shown that nascent autophagosomes recruit and load up the dynein-SNAP associated protein (SNAPIN) motor-adaptor transport machinery through fusion with late endosomes. Such a mechanism is critical for returning autophagic cargos to the soma for lysosomal degradation [50]. Besides, endocytic adaptor AP-2 is also reported to regulate retrograde transport of autophagosomes through direct interaction of AP-2α_A_, a large brain-specific subunit of AP-2, with LC3 or of AP-2β with the p150^Glued^ subunit of the dynein motor cofactor – the dynactin complex [69]. In addition, multiple dynein effectors such as the Striatin-interacting phosphatase and kinase complex, c-Jun N-terminal kinase–interacting protein 1 (JIP1), Huntingtin, and Htt-associated protein 1 (HAP1) have been proposed to play a role in the retrograde movement of autophagosomes [63-65]. Whether autophagosome retrograde transport in axons is dominantly controlled by a single consolidated, multi-component mechanism or comprehensively modulated actions through multiple adaptor and effector proteins of dynein motors remains to be scrutinised. Indeed, a recent study proposed an interesting model by which dynein effectors sequentially regulate autophagosome motility in axons, thereby linking autophagosome transport to its maturation for cargo clearance [68]. Specifically, JIP1 was shown to participate in initiating autophagosome transport in distal axons, whereas HAP1 and Huntingtin-mediated modulation of autophagosome transport mostly occurred in the mid-axon. Besides its role in axonal lysosomal transport [70,71], this study has further revealed that JIP3 is associated with autophagosomes and selectively regulates the transport of matured autophagosomes as autolysosomes [68]. Given that alterations in JIP1 and JIP3 have been implicated in AD [71-73], future work on their roles in AD-linked autophagy pathogenesis may provide new insights.

#### Autophagic cargo clearance

Autophagy is a lysosome-dependent pathway, and thus, lysosome-mediated cargo clearance is a limiting factor in autophagy activity [10,56]. Multiple lines of evidence have established that degradative organelles, including lysosomes and autolysosomes, are mainly located in the soma of neurons, the central site for autophagic cargo clearance [48,51,71,74-79]. As the primary catabolic compartment, lysosomes receive and degrade biomacromolecules from the secretory, endocytic, and autophagic pathways through the concerted action of acidic hydrolases [80,81]. Therefore, lysosomes need continuous replenishment of newly synthesised hydrolases to maintain their degradative capacity. Our previous work has revealed that efficient autophagy-lysosomal function requires proper retrograde transport of late endosomes, which are loaded with hydrolases [74,82]. Indeed, defects in this transport process trigger lysosome functional deficits due to trafficking impairment of essential hydrolases to lysosomes in the neuronal soma, resulting in abnormal accumulation of autophagic cargoes. In line with this work, a recent study has further uncovered that lysosomes with full activity of hydrolases are restricted from myelinated axons in mature, intact mouse brains [83]. Interestingly, besides autophagosomes and late endosomes, many notably tubulovesicular structures containing both lysosomal hydrolases and the Trans-Golgi network protein marker as Trans-Golgi network-derived transport carriers are present in these axons. The transport carriers facilitate the delivery of lysosomal constituents to axons, which are upregulated in the dystrophic axons of diseased neurons. However, the degradative capacity of organelles bearing retrograde motility in axons is relatively limited, and lysosomal proteolysis still mainly occurs in the soma [83]. Therefore, these findings support the notion that a nascent autophagosome in distal axons has a prolonged lifespan before encountering a lysosome, even though lysosomes can rapidly eliminate autophagosomes in the neuronal soma. Furthermore, the vulnerability towards axonal degeneration/dystrophy linked to neurodegenerative disorders could be attributed to a limited degradative capacity in axons owing to the restricted entry of mature lysosomes into axons.

### Autophagy mechanism in AD neurons

AD is characterised by abnormal accumulation of various forms of soluble and aggregated species of Aβ and Tau proteins in affected neurons. An enormous body of work has established that both Aβ and Tau can be degraded within lysosomes after being targeted by autophagy [56,84,85]. However, autophagy defects have been implicated in AD pathophysiology [56,85-87] (Figure 2). While Aβ is an autophagy substrate and the cellular levels of APP and Aβ are found to be partially regulated by autophagy [88], the interplay between Aβ and autophagy is complex, and the exact role of autophagy in the pathogenesis of AD remains poorly understood. Autophagy enhancement has been shown to reduce Aβ levels in different model systems [88-93]. Conversely, many studies in mouse models and cell culture have also revealed that autophagosomes, enriched with APP and Aβ-generating machinery BACE1 and PS1, function as an important site for amyloidogenic processing of APP and Aβ generation [89,94-99]. In addition, autophagy may also play a role in the extracellular secretion and deposition of Aβ [95,100], reducing intraneuronal Aβ accumulation. Indeed, AD mouse brains with loss of neuronal *atg7* exhibited a significant increase in intracellular Aβ levels accompanied by attenuation of extracellular amyloid plaque burden [95], suggesting that autophagy may mediate the secretion of Aβ and promote amyloid deposition in AD brains. A growing body of evidence has revealed some features of the secretory autophagy pathway that can be activated upon cellular stress, lysosomal inhibition, or activity-induced synaptic remodelling [101-103]. Thus, further investigations are needed to gain mechanistic insights into the role of secretory autophagy in Aβ secretion.

Despite the possible involvement of autophagy in the pathogenesis of AD, accumulating evidence indicates that defects affecting multiple steps of the autophagy-lysosomal system in AD contribute to neurodegeneration. Based on the data from the studies of BECN1 and phosphatidylinositol-binding clathrin assembly protein (PICALM), AD-related autophagy dysfunction could result from defects in autophagosome formation and maturation. Becn1 is a mammalian homolog of yeast Atg6 and an autophagy-related tumour suppressor gene [104]. AD brains exhibit a reduction in the levels of BECN1, which is caused by enhanced caspase 3 cleavage [96,105,106]. More importantly, genetic downregulation of *Becn1* in mice leads to neuronal autophagy deficits, Aβ accumulation, and neurodegeneration, suggesting neuroprotective effects of *Becn1* against AD-related pathologies [96]. Aside from these studies, BECN1 was also shown to directly interact with APP via an evolutionarily conserved domain and thus facilitate APP trafficking from the cell surface to autophagosomes [107]. Hence, defects in such a mechanism may also contribute to autophagy failure in AD. PICALM is a clathrin adaptor protein required to endocytose SNAREs [108]. Prior work has demonstrated that PICALM colocalizes with APP during endocytosis and regulates Aβ plaque formation in mouse brains [109], suggesting a possible role of PICALM in AD pathogenesis. Indeed, various single nucleotide polymorphisms and abnormal cleaved forms of PICALM were found in AD, but the mechanism responsible for such changes in PICALM remains unclear [110-113]. Furthermore, PICALM was proposed to regulate early formation and maturation of autophagosomes, implying that PICALM dysfunction may trigger autophagy defects in AD through such a mechanism [108]. Additional evidence indicates that PICALM acts as an autophagy receptor that interacts with LC3 by which PICALM mediates APP sequestration within autophagosomes, which could also be disrupted in AD. Combined, these lines of evidence suggest that autophagy dysfunction in AD neurons may be attributed to defects in autophagosome biogenesis, cargo sequestration within autophagosomes, and autophagosome maturation.

Defects in retrograde axonal transport of autophagosomes could also cause autophagy dysfunction in AD neurons. A large amount of evidence has demonstrated that AD brains and cellular models are characterised by the massive accumulation of autophagosomes and amphisomes along with other membranous organelles, such as LAMP1-positive endolysosomal organelles within dystrophic neurites and synaptic terminals [48,75,114-120]. Many studies have established that retrograde transport is essential for returning autophagosomes to the soma for clearance and thus prevents autophagic accumulation in distal axons. Therefore, abnormal autophagosome retention in the dystrophic axons of AD neurons suggests the possibility of retrograde transport deficiency. Indeed, our work has shown that robust autophagic accumulation at the presynaptic terminals of AD neurons is due to impeded retrograde transport of autophagosomes, which is caused by Aβ burden-induced disruption of dynein motor-Snapin coupling [117,121]. We have further demonstrated that BACE1 is concentrated within autophagosomes [98]. As a result, defective retrograde transport and, thus, abnormal accumulation of autophagosomes in AD axons and synapses intensify Aβ generation through the autophagy pathway. In addition to defects in Snapin-dynein motor-driven retrograde transport of autophagosomes, some studies also indicated a possible involvement of AP-2 in autophagic BACE1 accumulation in AD neurons, likely by impairing AP-2-mediated autophagosome retrograde movement. AP-2 was previously reported to regulate synaptic vesicle reformation and retrograde transport of brain-derived neurotrophic factor/TrkB receptors [69,122]. In agreement with the observation that induced pluripotent stem cell (iPSC)-derived neurons from patients with LOAD exhibited a decrease in AP-2 levels, abnormal BACE1 accumulation within autophagic vesicles and enhanced Aβ generation were also found in mouse brains lacking AP-2 [123]. However, the mechanism underlying AP-2 deficiency in AD neurons remains unclear. These findings imply that retrograde transport defects also contribute to autophagy dysfunction in AD, augmenting amyloidogenesis in axons and synapses. It is conceivable that retrograde transport defects-induced autophagosome accumulation may also promote Aβ release.

Lysosomal dysfunction, one of the primary cellular defects in AD, has also been shown to play a pivotal role in AD-related autophagy deficits [56,124]. Importantly, mutations in the *PS1* gene, the most common cause of fAD, have been found to disrupt lysosomal function and autophagy and lead to a robust acceleration of disease onset and neuropathological severity [48,125]. Apart from its γ-secretase activity, PS1 was found to regulate lysosomal acidification and protease activation by controlling the assembly of lysosomal vATPase. Defects in such a mechanism cause lysosome failure and autophagic clearance deficiency [126-128]. It is noteworthy that autolysosomal acidification defects were recently proposed as a primary driver of AD pathogenesis, as evidenced by the emergence of such defects well before extracellular amyloid deposition in AD [119]. The involvement of PS1 in the regulation of autophagy-lysosomal function was further supported by the evidence showing that PS1 controls the activity of the transcription factor EB (TFEB). In the nucleus, TFEB binds to the coordinated lysosomal expression and regulation (CLEAR) genes [129,130]. These genes encode for various proteins that can be grouped into distinct categories, such as lysosomal hydrolases and accessory proteins, lysosomal membrane proteins, and proteins involved in lysosomal biogenesis and autophagy [130,131]. PS1-deficient neural stem cells (NSCs) displayed decreases in TFEB and the transcriptional levels of autophagy-related genes [132]. In addition, loss of PS1 function was also found to alter local Ca^2+^ signalling and hamper retrograde movement of autophagic vesicles [133]. Combined, these results are the earliest demonstrations directly linking AD-causing genes to autophagy dysfunction and suggest one of the clearest examples of the rescue of autophagy failure in affected AD neurons through the correction of PS1 dysfunction-induced lysosomal acidification deficits. In addition, our work has revealed that lysosomal proteolysis deficits in AD could also be the result of impaired retrograde transport of late endosomes, which disrupts retrograde trafficking of retromer necessary for the trafficking of essential hydrolases to lysosomes. Thus, lysosome hydrolase deficiency impairs lysosomal proteolytic activity for autophagic cargo clearance in AD neurons [78]. Importantly, other studies have provided direct evidence showing that blocking axonal transport of LAMP1-positive endolysosomal organelles sufficiently triggers an increase in Aβ42 production in human iPSC-derived neurons without overexpression of mutant APP or PS1 and augments plaque pathology in a mouse model of AD [70,71]. In addition, recent work has uncovered that neuronal lysosomal phospholipase D3 mediates aberrant endolysosomal vesicle accumulation and promotes neuritic plaque development. Such effects correlate strongly with amyloid pathology and cognitive dysfunction in AD [134,135]. These findings collectively highlight a crucial role of lysosomal defects in autophagy failure and AD-related pathologies. Taken together, multiple mechanisms could be responsible for autophagy deficiency in AD neurons, including defects in autophagosome formation and maturation, retrograde transport deficits, and lysosomal dysfunction.

Hyperphosphorylation and aggregation of microtubule-associated tau (MAPT/Tau) is one of the hallmark pathologies of AD and a defining feature of a group of neuronal disorders termed tauopathies, including progressive supranuclear palsy, corticobasal degeneration, and frontotemporal dementias (FTDs) [136]. Many studies have established that pathogenic Tau is degraded within lysosomes after being targeted by autophagy [137-141]. Notably, aside from the build-up of neurofibrillary tangles (NFTs) as a key neuropathological feature of AD and other tauopathies, pathological Tau is also highly concentrated at synapses and plays a central role in perturbing synaptic function [142-152], suggesting a critical role of autophagy failure in the build-up of Tau and Tau-mediated toxicity. Earlier work reported abnormal accumulation of Tau-loaded autophagosomes in the brains of AD and other tauopathies [153]. In cultured neuronal cell lines, pathogenic Tau expression was also shown to disturb autophagy function, but the underlying mechanism was unclear [154].

A recent study has revealed that ring finger protein 216 (RNF216, also known as TRIAD3A) expression is decreased in AD patient brains. TRIAD3A is an E3 ubiquitin (Ub) ligase of the RING-in-between-RING class that functions as an autophagy adaptor and utilises E3 ligase and liquid-liquid phase separation to target Tau for autophagic degradation [155]. More importantly, overexpression of TRIAD3A reduces the accumulation of phosphorylated Tau levels in tauopathy mouse brains. Thus, the findings from this work indicate that autophagy deficits in the tauopathy of AD are likely caused by defects in the capture and sequestration of pathological Tau within autophagosomes. In addition, abnormal Tau appears to curb axonal transport by disrupting the dynein-dynactin complex, which could result in autophagosome accumulation and thus autophagic Tau clearance defects [156,157]. These results support a link between defective retrograde transport and tauopathy-related autophagy dysfunction. Of note, autophagy was implicated in Tau release, which was enhanced upon autophagy induction by starvation or pharmacological agents or upon lysosomal deficits but reduced under autophagy inhibition [158-161]. Thus, Tau-loaded autophagosomes in axons could be redirected for secretion through direct fusion with the plasma membrane and tauopathy-linked axonal autophagic accumulation further indicates defects in such a mechanism. Together, data obtained from all these studies firmly suggests that multiple mechanisms may underlie autophagy defects related to the tauopathy of AD.

Mounting evidence indicates that lysosomal defects also contribute to autophagy dysfunction and exacerbate Tau pathology in AD and other tauopathies. While lysosomal stress was shown to induce Tau accumulation within acidic degradative organelles and promote cleavage and neurotoxicity of Tau in mouse brains [162-164], recent studies have also uncovered that pathological Tau alters the endolysosomal system and blocks lysosomal degradative capacity by inhibiting nuclear translocation of TFEB [165,166]. In accord with these findings, upregulation of TFEB in tauopathy mouse models leads to a marked reduction in soluble phosphorylated Tau and insoluble Tau aggregates, mitigating cognitive impairment [167,168]. Moreover, pathological Tau was reported to bind to lysosomal membranes and thus perturb lysosome permeability *in vitro* and *in vivo* in an AD mouse model [138,169]. These results are consistent with the observation that lysosomal membrane integrity was disrupted in AD patient brains [170]. Additional lysosomal mechanisms in tauopathy-related pathologies have also been demonstrated. For example, the lysosomal exocytosis of Tau was recently proposed, and its loss of function has been found to augment Tau pathology [171]. These results collectively suggest that lysosomal disturbance may also occur at multiple steps and play an essential role in autophagy dysfunction associated with tauopathy.

## Autophagy of glia in AD

### Introduction of glias

Within the brain, there are two major neural cell types, neuron and glia. Glia make up most neural cells and have many roles to support and maintain normal brain functions. Originally, glial cells are thought of as secondary to neurons, only serving to support neuronal functions but later studies have proven glia’s role in neurotransmission, energy supply, regulation of the blood brain barrier (BBB), circuit formation, immune response as well as others [172]. In this section, we will discuss the functions of autophagy in three main types of glia; microglia, astrocytes, and oligodendrocytes; each having their own role in brain development, ageing, and AD. Microglia are the resident immune cells of the brain and serve several critical functions, including facilitating immune responses, brain surveillance, regulating neuroinflammation, and maintenance of neurogenesis and synaptic pruning. Microglia differ from other types of glia because of their myeloid origin. These cells arise from erythro-myeloid progenitors of the embryonic yolk sac and then migrate to the brain during early development in mouse embryo [173]. Astrocytes are the most abundant type of glia within the central nervous system (CNS), earning their name from their “star” shaped cell bodies. Astrocytes are crucial for the nutrients support to neurons, the synaptic formation and connection, and the formation and plasticity of neural circuits. Astrocyte’s endfeet is an essential component of the BBB for the removal of toxins and wastes [174]. During development, astrocytes develop from NSCs, or radial glia, in the ventricular zone and subventricular zone of the lateral ventricle. In mice, the generation of astrocytes starts at embryonic day 18, the time after neurogenesis is completed, and lasts until postnatal day 7. This switch is facilitated by transcription factors SRY-Box Transcription Factor 9 (SOX9) and Nuclear Factor I A, which induce gliogenesis and are implicated in astrocyte migration and maturation [175]. Single-cell RNA-sequencing from mice has shown that based on different gene expressions, astrocytes localised at specific brain regions can be clearly divided into subpopulations to serve different functions [176]. Among the markers of astrocytes, glial fibrillary acidic protein (GFAP), S100β, Aquaporin4, and connexins 30 are commonly used to identify their location and reactivation status. Another major glial cell type is oligodendrocytes. These cells are responsible for all the myelination that occurs in the CNS and maintenance of the myelin sheath which will need to be modified throughout all stages of life. Along with this, they also aid in the metabolic support of neurons as well as maintaining the integrity of axons [177]. Oligodendrocytes arise from NSCs-derived oligodendrocyte precursor cells (OPCs) and continue to differentiate into adulthood [178]. OPCs bound for gliogenesis express SOX9 early on but as they develop, they lose the expression of SOX9 [179]. OPCs constitute the most proliferative cells in the brain at homoeostatic stages, being evenly distributed throughout. As well as their ability to differentiate into oligodendrocytes, OPCs have the potential to become Schwann cells and astrocytes under certain conditions [180-182]. OPCs are closely associated with neurons, helping to form excitatory and inhibitory synapses and assisting in signalling [183]. The populations of additional glial cells including border-associated-macrophages, ependymal cells, pericytes, peripheral Schwann cells, and others that will not be discussed in this review as the autophagy function of these cell types has yet to be seriously explored in development and in AD.

### Autophagy of glia in brain development, homoeostasis, and ageing

#### Microglia

Microglia serve many functions in embryonic and postnatal brain development, including the regulation of neurogenesis, debris clearing, synaptic pruning, neuronal plasticity, circuit formation, and maintenance of brain homoeostasis [184,185]. Microglial autophagy is important in synaptic pruning. Kim et al., showed that the deletion of *atg7* in microglia causes dysregulation of synaptic abundance leading to problems with brain connectivity and cognitive impairment, resembling symptoms of autism spectrum disorder [186]. Adult microglia take surveillance of the brain microenvironment, looking for danger signals to eliminate potential threats. Under normal circumstances, microglial autophagy in the adult brain is responsible for the removal and degradation of misfolded proteins or damaged organelles. Once an injury or disease is detected, microglia launch into response to become activated; changing into amoeboid shaped morphology, proliferating, recruiting other immune cells, and releasing inflammatory cytokines. Autophagy in microglia is necessary for their proper immune responses. *Atg7* deficiency in microglia reduces the expression of immune and inflammatory response genes, limiting microglia’s ability to respond to stimulus [187]. After injury, it is necessary to limit the expression of proinflammatory cytokines because prolonged inflammation causes damage to brain functions. Accordingly, the deletion of *becn1* in microglia causes a sustained increase in the expression of proinflammatory genes like *nos2, nlrp3*, and *cybb* after injury, negatively affecting brain recovery [188]. Autophagy negatively regulates the inflammatory protein mitochondrial antiviral signalling (MAVS) through direct binding of LC3 to its LIR motif Y(9)xxI(12) [189]. These data support the role of autophagy in controlling the reactivity of microglia and their neuroinflammation status, which are also prominent in the brain of AD patients. In addition to autophagy’s role in controlling neuroinflammation, autophagy in microglia is also important to manage metabolism. This can be seen through the deletion of *atg7* in microglia with intracellular lipid droplet accumulation as well as increased levels of APOE [190]. Interestingly, the energy metabolism of glycolysis and oxidative phosphorylation in mitochondria of *atg7* deficient microglia experiences little change [190]. It is generally understood that as the brain ages, the levels of autophagy decrease, ultimately impacting glial cells’ ability to maintain homoeostasis and neuronal integrity. A subpopulation of microglia with activated ERK1/2 appears with age and they are dependent on canonical autophagy for survival. This population, called “ADAM”-Autophagy Dependent Age-acquired Microglia, upregulates canonical autophagy genes in ageing. Losing this population of microglia leads to neural cell death and increased mortality in autoimmune neuroinflammation [191].

#### Astrocytes

Autophagy is necessary for astrocyte differentiation in postnatal brain development. Downregulating *atg5, atg7*, and *p62* inhibits astrocyte differentiation from mouse NSCs [192-194], which give rise to glial progenitors for gliogenesis after birth. Nevertheless, another report indicated that the deletion of autophagy gene *rb1cc1* increases astrocytes number as the expense of reduced neurogenesis from postnatal NSCs [195]. This discrepancy in astrocyte differentiation may be through the non-canonical functions of Rb1cc1 in postnatal NSCs [196]. As a central node for catabolism, autophagy allows astrocytes to have resiliency to stress conditions of nutrient deprivations [197]. Compared to neurons, astrocytes have a higher level of autophagy activation when put under a starvation condition [198]. Astrocytes aid neurons with transcellular mitophagy, through which neuronal mitochondria can be transferred from axons to astrocytes through contact points, then being degraded by autophagy [199,200]. These protective mechanisms of autophagy in astrocytes support their functions in maintaining high metabolic rate for normal brain functions. Astrocytes could engulf protein aggregates and cell debris for brain homoeostasis. Facilitated by C4b opsonisation, astrocytes phagocytose and degrade microglia fragments through non-canonical autophagy of LC3-associated phagocytosis [201]. Autophagy is also modulated in astrocytes affected by Alexander disease, a neurological disorder caused by the rare mutations in human *GFAP* gene, the products of which constitute the majority of aggregated Rosenthal fibres within astrocytes [202,203]. Under these conditions, astrocytes activate autophagy to degrade mutant GFAP and avoid the accumulation of misfolded GFAP [204,205]. Activation of autophagy also potentially compensate for the lower proteasome activity to remove oligomer GFAP in this disorder [206]. In ageing, astrocyte’s functionality changes along with impaired autophagy activity. Aged astrocytes in humans brain experience atrophy in their morphology and have more dysfunctional mitochondria [207], suggesting defective mitophagy in old human astrocytes. In old mice, a unique astrocyte subtype exhibits dysregulated autophagy and morphology in the hippocampus. These astrocytes are termed “APDAs”-AutoPhagy Dysregulated Astrocytes. In these abnormal astrocytes, autophagosomes accumulate in swollen processes with impaired protein trafficking and secretion [208]. These aberrant changes in autophagy affect astrocyte’s ability to connect to and support neurons in old animals.

#### Oligodendrocytes

New oligodendrocytes continue to be generated from OPCs throughout adulthood. Deficiency of autophagy essential genes *atg5, atg9a*, or *becn1* in OPCs reduce their survival rate and affect their differentiation into mature oligodendrocytes for myelination [209,210]. Maintaining myelin sheaths is an essential function of mature oligodendrocytes and failure to keep intact myelin has been shown to contribute to the dysregulation of brain homoeostasis and may lead to diseases. Mitophagy receptor *bnip3l* (BCL2/adenovirus E1B interacting protein 3-like)-mediated mitophagy is required for the remodelling of mitochondria during the differentiation of oligodendrocytes [210]. Aber et al. revealed the roles of autophagy in myelination by deleting *atg7* in mature oligodendrocytes [211]. They found that *atg7* deficiency in oligodendrocyte causes increased myelin thickness and axonal abnormalities, leading to glial and neuronal cell death in affected animals. Similar to *atg7, atg5* deficiency in oligodendrocyte causes perturbed myelin maintenance and morphological defects in myelin membrane such as decompaction to induce increased axonal degeneration [212]. Deficiency of *atg5* in oligodendrocytes has also been shown to cause issues with motor function and neuronal loss in ageing mice [211]. These studies indicate the essential functions of autophagy in OPCs and mature oligodendrocytes for their functions in maintenance of myelin integrity in the CNS.

### Glial autophagy in AD

Glias have been implicated in the progression of neurodegenerative diseases and injuries, having roles that are both beneficial and detrimental to the brain [213]. Recent studies have begun to link the functions of glial autophagy to limit neuroinflammation and to clear pathogenic protein aggregates and damaged organelles in AD. The causative mutations of fAD gene APP, the major LOAD risk gene of Apoe polymorphism ε4 (Apoe4), and aggregated hyperphosphorylation of Tau in NFTs in AD brain have been associated with autophagy in *in vivo* and *in vitro* models. Most studies have focused on the impacts of these AD mutations and pathological changes on neuronal autophagy in the brain (*please see previous neuronal autophagy section for details*). Nevertheless, it is becoming clear that the AD mutations also have robust effects on glias, in some cases, which potentially are the drivers for AD onset and progression [214,215]. In this section, we will describe the impact of AD mutation products on autophagy in glias, followed by the discussion of mechanisms and functions of autophagy in glias for AD progression.

### Impact of pathogenic proteins in AD on glial autophagy

#### Aβ plaques

Aβ is the cleaved product of APP from BACE1 and γ-secretase, which create proteins that are usually 40 or 42 amino acids [216]. *APP* is one of the first identified fAD genes and there are approximately 25 pathogenic APP mutations causing fAD [217]. The glial mechanism underlying the effect of APP mutations on formation of Aβ plaques is still not very clear. Exposure to mutant APP and Aβ aggregates reduces autophagy in hippocampal neurons [218,219]. However, in culture, the acute accumulation of Aβ triggers higher autophagy activity in astrocytes [220] and in microglia [221]. Interestingly, long-term exposure to Aβ aggregates decrease the autophagic functions in microglia [222], meaning that as disease progresses, microglia are no longer able to remove this misfolded proteins through autophagy. In OPC, the presence of engulfed Aβ peptides stimulates the autophagic pathway for their degradation [223]. These studies imply the response of autophagy in glial cells to limit Aβ spreading at the onset of AD. The long-term impact of Aβ mutations on glias *in vivo* needs further investigation as they might exhibit different effects on glial autophagy in AD progression.

#### Apoe4

APOE is one of the major lipid binding proteins in the brain. The main source of APOE is from astrocytes and to a lesser extent from microglia in the brain under physiological conditions [224]. APOE transfers lipids and cholesterol generated from astrocytes to neurons via different cell-surface receptors for the membrane integrity and synaptic plasticity of neurons [225,226]. APOE is involved in energy metabolism and essential energy support for neurons, dysfunctions in these processes contribute to severe neurodegeneration in AD [227]. The biallelic *Apoe e4/e4* mutation is the strongest genetic risk factor for the development of LOAD [228-230]. APOE4 has been linked to the disfunction of autophagy in the brain. The APOE4 protein specifically binds the “CLEAR” element in the promoters of several autophagy genes to block their transcription [231]. APOE4 significantly decreases the expression and transcription of regulatory genes for autophagy, including FoxO3a, as well as mitophagy genes in human astrocytes [232,233]. In transgenic mice expressing human APOE3 and APOE4, astrocyte mitochondria are strongly affected by APOE4 to reduce mitophagy and mitochondrial fission with compromised mitochondrial functions in normal and stress conditions [234]. Homozygous expression of human APOE4 in astrocytes impair autophagy to reduce the effectiveness at removing Aβ plaques in APP/PS1 mice [235]. Like in astrocytes, APOE4 exacerbated mitophagy dysfunction and neuroinflammation without overt effect on bulk autophagy in cultured microglia [236]. The impaired mitophagy might be responsible for defective immune response of APOE4 microglia to Aβ accumulation to limit microglial activation and altering gene expression [237]. Recent findings indicated that APOE4 causes lipid metabolism dysregulation to accumulate lipid droplets in AD brains [238], which suggest defects in lipophagy (a selective form of autophagy to degrade lipid droplets) in APOE4 microglia. Taken together, these studies indicate that APOE4 in general impairs autophagy and specifically mitophagy machinery in glia and dampens their functions in elimination of Aβ plaques to accelerate AD progression.

#### Tau

Other than APOE4 and Aβ, NFT in neuron is one of the most significant pathological hallmarks of AD. Tauopathies are closely associated with neuroinflammation and activation of microglia/astrocyte. Aggregated form of the Tau-derived PHF6 peptide induces autophagy and activation of NLRP3 in human microglia [239]. The increased microglial autophagy helps to remove and degrade Tau tangles in AD brain. Astrocytes also play a role in the development of Tau tangles. Astrocytes can take up Tau fibrils through heparan sulphate proteoglycans-mediated micropinocytosis and engulf Tau monomers through a heparan sulphate proteoglycans-independent pathway. Upon internalisation into astrocytes, astroglial TFEB promotes Tau degradation and inhibit its transmission in tauopathies [240]. With decreased autophagy, it is expected that the Tau accumulation in astrocytes of dentate gyrus causes dysfunctions of mitochondria, impaired neurogenesis and reduced neuronal density, ultimately leading to cognitive defects in AD mice [241]. Compared to the other two major glias, the impacts of Tau aggregation and NFTs on autophagy activity in oligodendrocytes and OPC are not clear.

### Glial autophagy in AD pathology

#### Microglia

Most studies of microglial autophagy in AD are through the loss-of-function approach for essential autophagy genes in transgenic AD mice. The generation and accumulation of Aβ plaques is phenomenal pathology in these models. Microglia could detect and respond to Aβ plaques to reactivate and aid in the degradation and clearance of Aβ plaques at the early stage of AD. *Atg7* cKO microglia reduces their ability to reactivate and increases Aβ burdens with more toxic diffuse plaques as well as more oligomeric Aβ in 5xFAD mice [242]. In the clearance of Aβ plaques, non-canonical autophagy of LC3-associated endocytosis (LANDO) in microglia plays a role to recycle Aβ receptors CD36, TREM2 and TLR4 to the plasma membrane. Microglia that are deficient of *atg5, becn1*, and *rubcn*, but not *atg14* and *rb1cc1*, are incapable to perform LANDO to degrade Aβ and to stop cognitive decline in 5xFAD mice [243]. Autophagy genes in microglia also play a role in Tau models of AD. The deletion of *atg7* in microglia causes a higher load of NFTs in PS19 mice, indicating that autophagy stops the formation and seeding of Tau tangles [190]. The TFEB signalling maintains lysosomal homoeostasis in microglia and plays a critical role in tauopathy of AD [244]. The elimination of pathogenic protein aggregates by autophagy in microglia is not limited to Aβ and Tau. The mutations of α-synuclein (α-Syn) have been implicated in Lewy bodies for neurodegenerations of Parkson disease (PD) and Lewy Body dementia [245,246]. Microglia could clear α-Syn aggregates through the process of autophagy [247]. Another notable feature of AD is the progressive loss of synapses, which is strongly related to cognitive decline in AD patients. Deletion of *atg7* in microglia decreases several synaptic proteins including SV2B and vGLUT2 in 5xFAD and PS19 mice [190,242]. At the early stage of AD, even prior to the tremendous accumulations of Aβ plaques and obvious loss of neurons in AD brain, the deletion of *atg5* in microglia inhibits the functions of NSCs to generate new neurons in the hippocampus of 5xFAD mice [248].

Neuroinflammation is observed surrounding senile plaques and affects neuronal functions in AD. Autophagy deficiency in microglia causes PD-like symptoms by accelerating inflammasome activation to produce proinflammatory cytokines [249]. Autophagy in microglia suppresses neuroinflammation in 5xFAD mice as LANDO-deficient microglia significantly increases the production of tumour necrosis factor α (TNF-α), interleukin 1 β (IL-1β), and interleukin 6 (IL-6) after Aβ treatment [243]. In another autoimmune and neurodegenerative disorder of multiple sclerosis, Berglund et al. found that *atg7* deficiency, and more specific the impairment of non-canonical autophagy, prevents animal recovery from experimental autoimmune encephalomyelitis by prolonging neuroinflammation [250]. The initial elevated immune response in autophagy deficient microglia might be helpful to limit injuries, however, uncontrolled inflammation eventually impairs degradation of Aβ plaques, fragmented myelin and other debris to cause neuronal death in brain with diseases. The selective form of mitophagy to remove damaged or excess mitochondria is involved in the regulation of neuroinflammation. As a quality control mechanism for mitochondria, mitophagy ensures that the cells have a healthy and functional population of mitochondria under normal and pathological conditions. Mutations of mitophagy genes, such as *prkn, pink1*, and *lrrk2* are all involved in the pathogenesis of familiar PD [251-253]. In an aged brain, the mitophagy process becomes less efficient at removing mitochondria, potentially leading to accumulations of dysfunctional mitochondria [254-256]. Fang et al. reported that in the brains of AD mice, the rate of microglial mitophagy decreases by 60% to accumulate damaged mitochondria [257]. By enhancing the mitophagy activity in microglia, a population of healthy mitochondria is preserved with functions for better energy regulation, increased phagocytotic activity to remove Aβ plaques, and suppressed neuroinflammation in an APP/PS1 mouse model [257].

**Figure 3.**
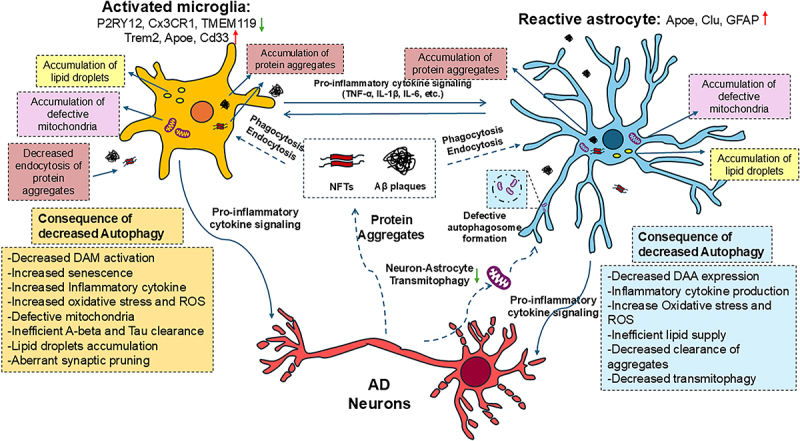
Autophagy of microglia and astrocyte in AD. In AD, microglia and astrocytes become activated with decreased gene expression of homoeostatic markers and increased expression for DAM markers or DAA markers, respectively. The protein aggregates of Aβ and NFTs from neurons will be engulfed and degraded by glial autophagy in early AD. As a response to protein aggregation, glial cells will produce and secrete pro-inflammatory cytokines, promoting neuroinflammation within the brain. In the context of autophagy deficiency, both microglia and astrocytes experience functional changes including decreased DAM/DAA activity, increases in inflammatory cytokine production, inhibition of lipid metabolism to accumulate lipid droplets, and increases in oxidative stress and ROS, as well as inability to degrade protein aggregates leading to severe AD phenotypes.

Recent studies revealed the heterogeneity of microglia in brain from developing, ageing, and AD brains, highlighting the diverse roles of microglia heterogeneity depending on the status of the brain [258-260]. With this, it has been demonstrated that a specific subtype of microglia defined as “DAM”-Disease-Associated-Microglia is associated with AD and other neurodegenerative diseases [258,261,262]. DAMs reduce the expression of homoeostatic markers P2RY12, CX3CR1, TMEM119 while they depend on TREM2 for their fully activation to protect AD brain. *Trem2, apoe* and *cd33* are DAM genes identified by single-cell RNA sequencing of microglia from AD model mice and mutations of these genes are AD risk factors in human genome-wide association study [263]. DAMs are engaged to Aβ plaques to reduce toxic Aβ accumulation, and it has been shown that the higher expression of DAM genes decreases the number of NFTs and improved cognition in AD individuals [264]. Trem2-deficient microglia or microglia expressing human AD-risk associated *TREM2* (e.g., *TREM2^R47H^*) display marked autophagy because of mTOR inhibition in brain of AD mouse model [265]. Increasing mTORC1 activity in microglia by specifically deleting tuberous sclerosis complex, upstream suppressor of mTOR, promotes the DAM population and improve cognitive outcome in AD mouse model [266]. Choi et al. discovered that DAM has a higher level of canonical autophagy genes than in other subpopulations of microglia isolated from 5xFAD mice. Furthermore, the deletion of *atg7* in microglia causes the downregulation of DAM genes, potentially affecting the microglia’s ability to respond to and engage with the Aβ plaques [242]. Further analysis of *atg7* cKO microglia identify their morphological changes to senescence, as well as altered expression of senescent genes *laminb1, γh2a*, and *hmgb1*. The removal of senescent microglia improved the condition of AD pathologies [242]. Senescent microglia with dysfunctional autophagy have also been implicated in the progression of PD [267]. The impact of autophagy deficiency in microglia on AD pathology is described in Figure 3.

Based on the data from the loss-of-function studies, boosting autophagy activity in microglia could reinforce their functions in neuroinflammation and neurodegenerative diseases. Indeed, increasing autophagy activity in microglia accelerates their transition from homoeostatic status into an anti-inflammatory M2 phase to alleviate neuroinflammation and potential damages [268]. Interferon gamma stimulation has been shown to increase the expression of autophagy genes *atg5* and *atg7* in microglia. Increased autophagy limits the spreading of Aβ burden in APP/PS1 mice, which could be attributed to the enhanced activities of phagocytosis or clearance of Aβ in microglia after Interferon gamma treatment [269]. Cannabinoid receptor 2 (CB_2_Rs) are expressed and upregulated in activated microglia surrounding senile plaques in AD patient’s brain and several agonists for CB_2_R alleviate the AD patients’ symptoms [270]. In mice that suffered from postoperative cognitive dysfunction, it is found that activating TFEB-mediated autophagy via CB2R improves their cognitive performance [271]. The promotion of TFEB activity causes the downstream effects to regulate lipid metabolism and to inhibit neuroinflammation associated with neurodegeneration [272]. These available data indicate that increasing the TFEB-CB2R-autophagy pathway and other autophagy related downstream signalling in microglia is a potential strategy to slow down or even halt AD progression.

#### Astrocyte

Astrocytes take on the roles of up taking and degrading Aβ accumulations and participating in neuroinflammation in AD. Through specific knocking down of *lc3b* and *p62* in astrocyte, the Aβ burden significantly increases in APP/PS1 mice [273]. This evidence supports the idea that autophagy in astrocyte limits the spread and intensity of Aβ plaques. Overexpression of autophagy protein LC3B in astrocyte delivered by adeno associated virus rescues Aβ plaques and improves cognitive function in AD mice [273]. Astrocytic deletion of clock gene *bmal1* reduces Tau and α-Syn pathology through upregulation of autophagy chaperone *bag3* to prevent protein aggregation. Overexpression of *bag3* in astrocyte reduces α-Syn spreading in mice [274]. Like in AD, astrocytes play an important role in the degradation of α-Syn in PD brain. After α-Syn released from PD neurons, it is up taken by astrocytes and removed through autophagy. Di Domenico et al. found that astrocytes derived from PD patient’s iPSC have dysfunctional autophagy and α-Syn accumulation in these cells, which in turn accelerate the neurodegenerative phenotypes [275]. αB-crystallin, a small heat shock protein, inhibits autophagy and causes further accumulation of α-Syn in astrocytes [276]. The autophagic breakdown of Aβ in astrocyte generates and provides aspartate and toxic ammonia to impact downstream urea cycle. The astrocytic urea cycle exerts opposing roles of beneficial Aβ detoxification and detrimental memory impairment in AD [277]. These studies indicate the complicated or even contradictory functions of autophagy in astrocytes for AD progression. In future studies, the specific manipulation of autophagy essential genes in astrocytes will provide critical information to understand their functions in AD.

Astrocyte is a major source for sustained neuroinflammation in neurodegenerative diseases. Astrocytes release cytokines after Aβ stimulation and this inflammatory response in reactive astrocyte is a factor in neuronal degeneration in AD [220]. Increasing autophagy through mTOR inhibition in astrocytes reduces the Aβ caused neuroinflammation [278]. Mitophagy is also critical to the functions of astrocytes as well as the stability of mitochondrial network to provide energy support to neurons. Astrocytes derived from hiPSC of FTD type 3 patients have dysfunctional autophagy function with the accumulation of damaged mitochondria and ultimately dysregulated energy metabolism [279]. In normal circumstances, astrocytes carry out transmitophagy [199] to uptake mitochondria exported from neurons to help in their degradation in autophagosome. Transmitophagy is significantly higher in astrocytes from human with AD and AD mouse model [200]. The function of astrocytic transmitophagy of neuronal mitochondria is important to the pathogenesis of PD. The uptake of neuronal mitochondria by astrocytes prevents the accumulation of damaged mitochondria in dopaminergic neurons and neuroinflammation in PD [280]. It is reasonable to expect that transmitophagy can perform similar protective functions in astrocytes to alleviate AD progression.

Like microglial heterogeneity, there has been a specific sub-population of astrocytes identified within the brains of AD mice, and these cells are grouped as “DAAs”-Disease-Associated-Astrocytes. DAAs share more similar expression of genes with reactive astrocytes compared to homoeostatic ones, while DAAs specifically express genes involved in endocytosis and ageing as well as amyloid metabolism and clearance, like *apoe* and *clu* [281]. The autophagy chaperone protein *BAG3* is also expressed in DAA-like cells from human AD brains [274]. DAA emerges during early AD before clinical symptoms present themselves [281]. Interestingly, mouse brains with AD show a significantly accelerated increase in APDAs, another type of astrocyte heterogeneity in ageing, suggesting potential roles for APDAs in AD-related cognitive decline and synaptic pathology [208]. The relationship between DAA and APDA is of great interest for future investigation. Senescent astrocytes contribute to AD through unknown mechanisms. Evidence has shown that senescent astrocytes have impaired mitophagy, with an accumulation of mitochondria within lysosomes. Modulating mitophagy rescues astrocytes from susceptibility to mitochondrial stress [255]. Addressing dysfunctional mitophagy and senescence-related changes in astrocytes as a promising approach for developing therapies to fight against AD. The impact of autophagy deficiency in microglia on AD pathology is described in Figure 3.

#### Oligodendrocytes

OPCs can uptake and degrade Aβ aggregates through autophagy though they are not as efficient as microglia [223]. As previously mentioned, having thicker myelin in autophagy deficient oligodendrocyte causes dysfunction in neurons, neurodegeneration, and glial cell death, which is like the circumstances found in AD [211]. Because of space limit and lack of expertise in oligodendrocyte, we will not discuss the detail mechanisms and functions of autophagy in oligodendrocytes and OPCs in this review. People who are interested in this topic can read the ref [282]. for more information about recent progress.

## Autophagy of brain endothelial cells in AD

### Blood-brain-barrier (BBB) and Brain endothelial cells (ECs)

BBB is a highly specialised structure and a biochemical barrier encompassing the microvasculature of the brain, which is essential for maintaining brain homoeostasis. The BBB comprises special endothelial cells, capillary basement membrane, astrocyte end-feet, and pericytes. Intercellular communication is essential for the development and function of the BBB, with each cell type playing a different role in BBB function. In this section, we will focus on the function of brain ECs that are part of the BBB and how they differ from ECs in the rest of the body, the role of autophagy in ECs and BBB, and how EC autophagy plays a role in AD.

The vasculature of the CNS during development begins when angioblasts enter the head region and form the perinuclear vascular plexus [283]. Then the perineural plexus invades the neuroectoderm via vascular sprouts, leading to angiogenesis [283]. The vessels continue to proliferate and form capillaries in the ventricular zone of the developing brain [283]. Angiogenesis occurs at a maximal rate in the early postnatal period and slows down with ageing [284]. As the brain vessels develop, they lose fenestration and become smaller [285], and the junctions between ECs become more interconnected [286]. Brain ECs present characteristics that differentiate them from ECs in the rest of the body, which have a flattened appearance, lack of fenestration as previously mentioned, higher expression of complex inter-endothelial tight junctions, fewer caveolae, lower levels of pinocytosis, higher number of mitochondria, and specific expression of transporters to maintain homoeostasis [287-291]. There are two receptors that are important for the differentiation of brain ECs. The first is the glucose transporter-1 (GLUT-1) which is an integral membrane hydrophobic protein that is highly expressed during embryonic development, and expression diminishes as maturation continues in most cells except in brain ECs in the BBB [292]. GLUT-1 expression is more prominent in the abluminal side of the membrane [293] as it provides glucose for energy production [292]. The second one is P-glycoprotein, an ATP-binding cassette transporter responsible for the efflux of many harmful compounds into the luminal side of the BBB [294]. Additionally, brain ECs highly express tight junction proteins, specifically occludin and members of the claudin family, including Claudin 1, 3, 5, and 12 [295].

### Autophagy in brain ECs and BBB

Autophagy in EC preserves redox homoeostasis, mitochondria quality control, and responses to inflammation, fulfilling vasculoprotective functions. While autophagy is typically activated to maintain homoeostasis, prolonged exposure to stress conditions can deregulate autophagy, which could be detrimental to EC function and promote autophagic cell death [296]. Shear stress caused by laminar flow, not oscillatory or low-magnitude flow, promotes autophagy in ECs in a reactive oxygen species (ROS)-dependent manner [297]. Steady shear stress increases the expression of BECN1 and LC3, established autophagosome markers [298]. Additionally, vascular endothelial growth factor (VEGF) activates the autophagy pathway *via* AMPK-dependent phosphorylation of ULK1 [299]. EC dysfunction is an early stage of atherosclerosis, in which one of the underlying mechanisms is increased production of ROS [300]. Lu et al demonstrated that TFEB overexpression in ECs reduces the concentration of ROS and inhibits inflammation *via* increased transcription of antioxidant genes in ECs [301]. TFEB overexpression activates AMPKα signalling and enhances autophagy in ECs, which promotes angiogenesis in mouse hindlimb ischaemic models [302]. Another group found that TFEB plays a role in modulating autophagy during angiogenesis through the transcriptional control of VEGF receptor 2 [303].

**Figure 4.**
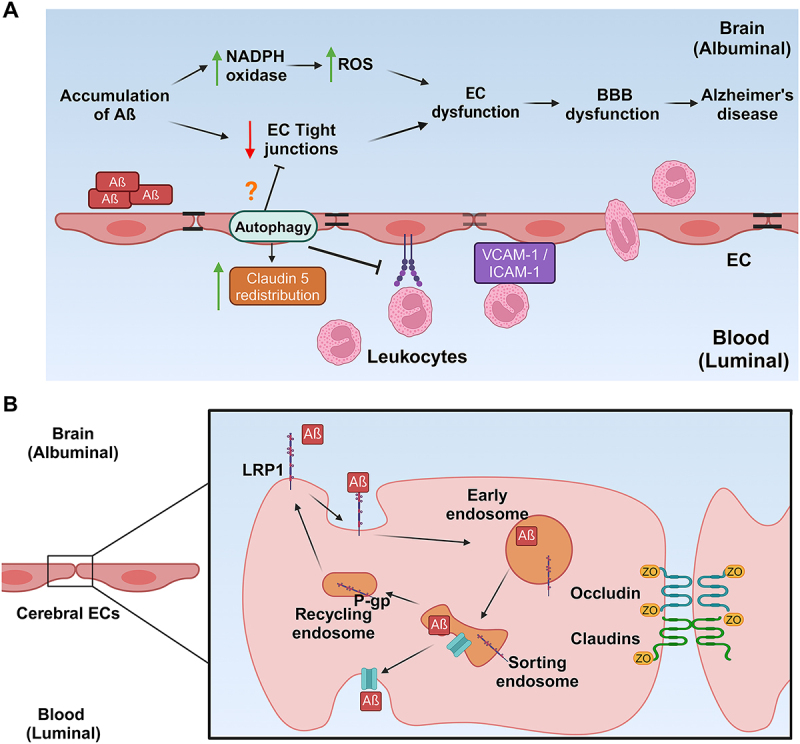
Schematic representation of autophagy in BBB in AD pathology and Αβ transcytosis in cerebral ECs. A. Aβ accumulation elevated mitochondrial NAPDH oxidase, leading to increased ROS. Elevated ROS and disrupted tight junctions (TJ) in ECs compromise BBB integrity and contribute to AD progression. In AD, inflammatory cytokines promote leukocyte infiltration by binding to proinflammatory adhesion molecules in ECs such as ICAM-1 and VCAM-1. This infiltration exacerbates brain inflammation and increases BBB damage. Autophagy increases the recycling of the TJ protein Claudin 5, thus improving barrier function in ECs. However, the role of EC autophagy in BBB dysfunction and AD pathogenesis remains unclear. B. Αβ clearance *via* transcytosis in cerebral ECs in the BBB. Αβ binds to LRP1, initiating endocytosis *via* PICALM. In the early endosomes, Αβ is dissociated from LRP1 and is sequentially secreted *via* P-glycoprotein into the bloodstream.

The exact role autophagy plays in the maintenance or disruption of vascular EC barrier remains to be elucidated, yet there are multiple studies that provide us with an insight into what this role might be. One study showed that inhibiting ECs’ autophagic abilities significantly induces ROS and increases the barrier permeability [304]. Amino acid deprivation impairs the endothelial barrier *via* decreased expression of tight junction proteins in intestinal endothelial cells [305]; a later study reinforced this principle in the BBB as it showed that serum deprivation reduces the expression of tight junction proteins, such as Claudin-5 (Cldn5), in the BBB and that inducing autophagy provides a protective role in maintaining BBB integrity [306]. Autophagy is enhanced in brain ECs with Akt-mTOR-p70S6K inhibition, protecting the integrity of the brain EC barrier from breakdown during early starvation induction [306]. These studies indicate that autophagy preserves BBB function as it is essential for the recycling of tight junction proteins (Figure 4A). However, other studies observed phenotypes where pathological conditions create autophagy-mediated cell damage or cell death [307-309]. Autophagy can have either protective or detrimental properties, which can be in part attributed to the cell types and the pathological conditions. Some studies demonstrated that autophagy could induce cell death under ischaemic damage in the brain [308,309]. Meanwhile, studies that demonstrated the protective role of autophagy focus on tight junction expression in intestinal ECs [305]. This highlights a major gap in knowledge about the role of autophagy in BBB that needs to be addressed.

### BBB dysfunction in AD

Αβ accumulates in the parenchyma of the neurons, preventing interneuron communication and restricting cerebral capillary blood flow [310]. The BBB responds to inflammatory stimuli like Αβ [311] by activating signalling pathways that promote the production of ROS and upregulate the proinflammatory secondary messengers. Exposure to Aβ increases the membrane permeability of EC [312], leading to elevated calcium influx into the mitochondria and subsequent activation of NADPH oxidase [313]. Aβ binds to the transient receptor potential melastatin 2 channel and elevates intracellular Ca^2+^ [314]. While we are beginning to understand how the accumulation of Aβ can trigger Ca^2+^ receptors, such as transient receptor potential melastatin 2, the exact mechanisms by which ROS increases BBB permeability remain unclear. A study showed that Αβ can directly upregulate the expression of metalloproteinases and receptor for advanced glycation end-products (RAGE) in mouse brain ECs [315]. Accumulation of Tau protein in the brain promotes the secretion of TNF-α and cytokine MCP-1 [316-318]. Particularly, when perivascular Tau accumulates in hippocampal blood vessels, it triggers BBB dysfunction [318]. Inflammatory mediators are highly secreted in AD patients [319]. Internal factors that can induce neuroinflammation include sex, age, and genetic factors [320]. Systemic inflammation can induce neuroinflammation through the BBB *via* IL-1β, TNF-α, and IL-6 [321]. These inflammatory cytokines can activate and alter the function of glial cells, activating microglia that, in turn, promote the secretion of more pro-inflammatory cytokines [321]. Infiltration of leukocytes related to both innate immunity and adaptive immunity has been seen in AD patients [322]. When leukocytes migrate from the blood into the brain, they must degrade the endothelial basement, which promotes BBB dysfunction [323]. The interaction between leukocytes and the vasculature in the CNS is mediated by endothelial P-selectin and E-selectin that interact with P-selectin glycoprotein ligand-1 on leukocytes. This interaction promotes the adhesion and migration of leukocytes to ECs *via* adhesion molecules, such as intercellular adhesion molecule-1 and vascular cell adhesion molecule-1, expressed on the surface of activated ECs [324,325]. The inflammatory leukocyte infiltration through BBB is illustrated in Figure 4A.

The BBB is a unit of multiple cell types; therefore, we must also acknowledge their role in BBB dysfunction. Chronic exposure of microglia to Αβ reduces its ability to clear Αβ, leading to further accumulation of Αβ [326]. The exposure also induces the expression of proinflammatory cytokines like TNF-α, IL-6, IL-1β, and NO [327]. Microglia also show upregulation of RAGE in AD, leading to prolonged activation of the microglia inciting the release of more proinflammatory cytokines [328]. This chronic inflammatory state exacerbates neuronal damage and facilitates AD progression. In a similar fashion, under pathological conditions, astrocytes increase the production and secretion of proinflammatory factors, such as cytokines and chemokines [329]. The elevated secretion of proinflammatory factors further increases ROS production *via* the activation of the AKT signalling pathway [330]. These factors induce BBB dysfunction in different ways, yet they all promote AD pathology [321]. Accumulation of Αβ in the vasculature of the brain, also referred to as cerebral amyloid angiopathy, prevents its drainage *via* the interstitial fluid, promoting neuroinflammation and increasing the production of ROS [331-333]. In addition to EC dysfunction, the dysfunction of pericytes [334] and smooth muscle cells [335] also contributes to increased BBB permeability, reduced barrier function, and disease progression in AD patients.

Smaller amounts of Αβ can be cleared through the bulk flow of the interstitial fluid, uptake by microglial or astrocytic phagocytosis, or transport across the BBB [336]. The BBB plays a role in the clearance of Αβ from the brain *via* transcytosis into the circulating blood. Αβ binds to cell surface receptors such as RAGE [337,338], scavenger receptor type A [338], and lipoprotein receptor-related protein 1 (LRP-1) [339-341]. Under basal conditions, Αβ binds to LRP-1, and the complex is internalised by PICALM-mediated endocytosis into early endosomes, where Αβ is dissociated from LRP-1 and secreted by P-glycoprotein into the blood [341]. The dysfunction of this brain-to-blood transport mechanism contributes to the accumulation of Aβ in the CNS (Figure 4B). At the same time, RAGE expressed on the luminal side of the BBB can uptake Αβ into the brain [342]. The expression of both LPR-1 and RAGE have been linked in AD patients. LRP-1 is downregulated, and RAGE is upregulated, leading to increased accumulation of Αβ in the brain [343]. After being secreted from the brain into the blood, Αβ binds to different proteins and can be degraded by other cell types, such as monocytes and erythrocytes [344]. The gene mesenchyme homeobox 2 may represent an additional vascular-specific pathway that contributes to the dysfunction of autophagy in AD. Wu et al. demonstrated that the ablation of mesenchyme homeobox 2 leads to aberrant angiogenesis, activation of proapoptotic pathways, and downregulation of LRP-1. These alterations can disrupt the integrity and function of BBB, exacerbating vascular contributions to AD pathology [345].

### Autophagy of BBB in AD pathology

Autophagy has been shown to be downregulated in AD patient’s post-mortem brains [153]. The 3xTg-AD mouse model also shows decreased protein levels of ATG7 and glycosylated lysosomal membrane protein 1, critical autophagy, and lysosome-related proteins [346]. Most of the current literature focuses on autophagy in neurons and more recently in microglia; thus, the role of autophagy in ECs remains to be fully defined. A study on human brain ECs demonstrated that the accumulation of Αβ inhibits the proliferative activity of ECs by inducing “self-digesting” autophagy. This effect is mediated by inhibition of the AKT pathway, which is crucial for cell survival and proliferation [307]. Evidence demonstrating the importance of Aβ in the disruption of the BBB has been published, yet the exact mechanisms are to be determined [337-339]. Further studies are needed to provide better insight into the role of autophagy in ECs and other cell components of BBB in AD. Some areas of investigation include whether autophagy directly degrades and the mechanisms that could activate autophagy as an alternative to transcytosis for Aβ clearance through BBB. Understanding these processes may reveal new therapeutic targets to protect BBB function and enhance Aβ clearance, alleviating AD progression.

## Therapeutic values of autophagy in AD

Given that AD-associated accumulation and aggregation causes disease primarily via toxic gain-of-function mechanisms that lead to neurodegeneration and cognitive dysfunction [56], one way of combating disease involves lowering amounts of such toxic protein aggregates. Both Aβ and Tau are autophagy substrates, and accumulating evidence suggests that enhancing Aβ and Tau clearance by targeting autophagy induction is an attractive therapeutic strategy for AD. Not surprisingly, stimulating autophagy activity has drawn significant attention as this strategy reduces the levels of both the soluble and aggregated species of these proteins and has been associated with protective outcomes [347,348]. Many small molecules employed to potentiate autophagy have been validated in *in vivo* models and can be classified into two major groups for acting through mTOR-dependent or mTOR-independent targets. Rapamycin, a relatively selective inhibitor for mTORC1 [349,350], and its analogues have demonstrated benefits as autophagy enhancers that decrease Aβ and Tau levels, ameliorate neuropathology, and attenuate cognitive deficits in the animal models of AD [90,92,351-354]. Given the various roles of mTOR signalling, the main caveat with mTOR inhibitors is the possibility of off-target effects. Thus, mTOR-independent autophagy inducers could be a better choice. Trehalose, a disaccharide with pharmacological chaperone activity, is a widely studied autophagy enhancer in neurodegeneration models [20,355], and has been demonstrated to have therapeutic effects concomitant with autophagy enhancement in both cellular and animal models of AD [139,140,356,357]. Other mTOR-independent autophagy inducers, including metformin, Methylene blue, lithium, and resveratrol, enhance autophagy by activating AMPK and have been shown to alleviate disease phenotypes in AD models [358-362]. As expected, drugs affecting AMPK signalling can impact multiple processes besides autophagy, some of which might amplify or limit therapeutic efficacy [363]. Besides the compounds acting on mTOR-dependent and -independent targets, many non-small-molecule approaches have also been developed for autophagy enhancement. These mechanisms include activation of TFEB targeting genes, Beclin1 induction, SIRT1-coupled LKB1–AMPK*α*, TyrRS–PARP1–SIRT1, and Wnt–GSK3*β*–*β*-catenin signalling pathways [364]. For instance, the delivery of the TFEB gene has been able to reduce pathological Tau levels and mitigate cognitive dysfunction in tauopathy models [167,168]. Parkin overexpression also showed beneficial effects in an AD model, likely through the activation of mitophagy [365]. It is worth noting that, apart from these well-studied strategies for targeting autophagy, some alternatives have been developed, for example, by using molecular glues to link substrates to autophagy machinery such as LC3 to enhance targeted removal of substrates [348]. Our recent work has revealed that stimulating mitochondrial bioenergetics by supplementing anaplerotic amino acid glutamine to enhance anaplerosis can boost autophagy functionality for Tau clearance in the cellular and mouse models of tauopathy. Such effects are attributed to oxidative phosphorylation-enhanced mitochondrial biosynthesis and the associated augmentation of PE supply for autophagosome biogenesis. Furthermore, early stimulation of anaplerotic metabolism potentiates autophagy activity and attenuates Tau pathology, thereby counteracting memory impairment in tauopathy mice. Similarly, mitophagy inducers abrogate amyloid-β and tau pathologies, and improve the AD animals’ memory [366]. Thus, these findings support stimulation of anaplerotic metabolism as a new therapeutic strategy to prevent the build-up of pathological Tau in the neurons of AD and other tauopathy diseases [367].

Relative to autophagy induction, preventing or reversing lysosomal/autolysosomal dysfunction is less explored as a therapeutic target. Compelling results from disease models underscore the pathogenic importance of lysosomal dysfunction in AD and strongly support the potential of this approach. Genetic ablation of cystatin B, a lysosomal cysteine protease inhibitor, reduced β-amyloid pathology and mitigated cognitive defects in a mutant APP transgenic mouse model [368]. Similar beneficial outcomes have been seen in other APP mouse models with cystatin C deletion, cathepsin B overexpression [369], or pharmacological elevation of cysteine protease activities [370]. Given that defects in lysosomal acidification attributed to the lysosomal ATPase deficiency cause lysosomal failure in degradation of autophagic substrates in AD neurons [126,371,372], an approach of restoring lysosomal pH using poly(DL-lactic-co-glycolic acid) nanoparticles was developed and shown to rectify lysosomal deficits and autophagic degradation in neurons [373]. Therefore, re-acidifying lysosomes could be an effective strategy for boosting autophagy activity in treating AD. As described above, additional approaches for lysosomal enhancement exploit TFEB-mediated upregulation of the gene transcription that promotes autophagosome formation and lysosomal biogenesis. Indeed, overexpression of TFEB or elevation of TFEB nuclear translocation alleviates disease phenotypes in both APP and Tau mouse models [167,168,374-377]. Notably, recent studies have shown that stimulation of synaptic activity can protect against pathological Tau accumulation/aggregation via autophagy-lysosomal degradation by enhancing nuclear translocation of TFEB in cellular and mouse models of tauopathies, which provides encouraging support for the use of synaptic stimulation against autophagy and lysosomal defects in AD and other tauopathies [378,379].

The evidence discussed in this review has demonstrated that autophagy can promote clearance of the primary toxic, pathogenic entities and thus serves as a promising upstream therapeutic target for AD from the perspective of developing pharmacological interventions. However, the current knowledge remains limited on whether autophagy in neural and other specific cell types can be therapeutically manipulated in the intact brain. To make autophagy modulation an effective therapeutic approach for AD, future research needs to be focused on deciphering molecular details of autophagy regulation in different brain cell types (including neuron, glial cells, and EC) and exploring the strategy for targeting autophagy in a diversified and combined manner. It is also important to determine the therapeutic window for autophagy enhancer in AD treatment as the loss of neurons is irreversible in AD. Finally, identifying small-molecule effectors will facilitate research on whether autophagy can be enhanced to make neurons and other cell types more resilient to environmental toxins, cellular stressors, ageing, and genetic risk factors of AD. It should be kept in mind that these effectors will need to bypass BBB to achieve the maximal effect to boost autophagy in neural cells and in brain EC. Therefore, continued robust and comprehensive mechanistic investigations to advance our understanding of autophagy will tremendously benefit the future development of therapies to combat AD more effectively.

## Summary

Here, we summarise the functions and mechanisms of autophagy in major brain cells, including neurons, glias, and brain EC for the pathological changes in AD. We can expect that neurons are the focus of current and future AD research as they directly impact the symptoms, progression, and outcomes of this neurodegenerative disease. However, we already appreciate that studies in other types of brain cells not only help us understand the pathology of AD but also provide new therapeutic targets for effective treatments. In the future, more efforts need to be integrated to analyse autophagy in different brain cells and the cell-cell communication in AD brain, especially at the early stage of AD initiation. We want to emphasise that even though enhancing autophagy is a potentially powerful strategy for AD, if not controlled well, autophagic cell death might happen in neural cells in neurological disorders, for example, in hippocampus under ischaemia. This situation is like the double-edge-sword effect for autophagy inhibition in cancer therapy. Nevertheless, the development of targeted therapy, e.g., specific mitophagy inducer, might be able to overcome the unspecific effect of overactivated bulk autophagy to relieve the energy crisis of AD neurons. To better utilise the autophagy inducer/activator in AD prevention/treatment, the identification of novel biomarkers from blood, cerebral spinal fluid, and imaging for AD progression would be essential. Lastly, the role of autophagy in immune, lymphatic, and glymphatic systems for AD pathology and therapeutic value is not clear, which opens opportunities for autophagy researchers to explore these new fields.
